# Identification of metastatic cell nucleus in human prostate cancer by electron microscopy

**DOI:** 10.2144/fsoa-2019-0141

**Published:** 2020-07-07

**Authors:** Akhouri A Sinha

**Affiliations:** 1 Research Service, Minneapolis Veterans Affairs Healthcare System, Minneapolis, MN 55417, USA; 2 Department of Genetics, Cell Biology & Development, University of Minnesota, Minneapolis, MN 55455, USA; 3 Masonic Cancer Center, University of Minnesota, Minneapolis, MN 55455, USA

**Keywords:** cancer requires mutation, chromatin harbors mutated DNA/genes, electron dense DNA molecules, invasive and metastatic cell types, loss of nuclear membranes releases DNA/genes, mutation imparts proliferation advantage, nuclear plasticity is indicative of the metastatic cell

## Abstract

**Aim:**

Metastatic prostate cancer is responsible for a large proportion of deaths worldwide. The aim of this study was to identify metastatic cells and determine if stromal invasion by cancer cells differs from those during metastasis.

**Methods & results:**

Tissue biopsy/prostatectomy samples, visualized by transmission electron microscopy, identified that metastatic cells are a lineage of stem cells, which have dedifferentiated into cancerous columnar/cuboidal cells. These cells demonstrate nuclear plasticity; the loss of nuclear membranes and boundary between nucleus and cytoplasm; and the presence of electron dense molecules, which can readily pass through basement membranes and enter the capillary, ready for dissemination to metastatic sites.

**Conclusion:**

This is the first study to demonstrate differences between invasive and metastatic cell types.

## Lay abstract

Being able to identify metastatic prostate cancer at the initial point of diagnosis could result in earlier targeted treatment, which could extend the patients life. Here, we identified that the source of metastasis is dedifferentiated columnar/cuboidal cells, visualized through transmission electron microscopy. Additionally, these have morphologically distinct characteristics from invasive cancer cells. These include nuclear plasticity; the loss of nuclear membranes and boundary between nucleus and cytoplasm; and the presence of electron dense molecules. This is the first study to demonstrate differences between invasive and metastatic cell types.

A brief review of the vast literature on prostate cancer (PC) and its metastasis has demonstrated that metastasis in humans is distinctly different from that in animal models and cell lines [1–4]. Metastasis occurs in nearly every human solid organ cancer. It varies greatly in human cancer patients, for example, between prostate, breast, colorectal, glioblastoma and pancreatic cancers [4–6]. Mutation in DNA/genes of stem cells of the benign prostate can lead to the development of PC, as it can in benign organs resulting in other solid organ cancers [7,8]. Mutation imparts a chronic proliferative advantage to invasive and metastatic cancer cells, but not to the benign prostate or during benign prostatic hyperplasia (BPH) [9–12]. Many mutagens (such as pesticides, herbicides, toxins, chemicals, contaminated food and water) circulating in capillaries surrounding prostate glands have the potential to induce mutations in the genes of stem cells. Repeated exposures to mutagens can produce deadly cancers. Specific mutagens and the numbers of mutated genes are unknown in PC and other solid cancers [9,10]. These mutagens produce heterogeneous cancers [9,10]. In all, metastasis is responsible for approximately 10% PC deaths [13].

Invasive and metastatic cells require proteases to lyse the acinar basement membranes, capillary and lymphatic basement membranes to enter in general circulation. Previous studies have identified a variety of proteases (such as cathepsin B, plasminogen activator, metalloproteases) that are required for cancer cells to reach the prostatic stroma as invasive cells and to reach distant metastatic sites [2–6]. Proteases come from invasive cells, stromal cells, or both. We have reported on the morphology of stem cells [14–16]. Stem/invasive cells readily pass through the acinar basement membranes and colonize prostatic stroma. Proliferation of cancer cells leads to the pathological patterns described by Gleason grades [17,18]. Several authors have previously identified and measured nuclear shape and nuclear morphometry [18–20]. Invasive cells need to breach the capillary wall to enter the general circulation. They must exit the capillary wall before entering a distant organ (such as pelvic bones, liver, lungs, brain) to establish metastasis. This process also requires proteases for distant organ metastasis. Stem cells alone produce insufficient amounts of proteases to lyse capillary walls and enter general circulation and to exit from the capillary to metastatic sites. Prostatic columnar/cuboidal cells, lineage of stem cells, are dedifferentiated cancer cells [14,15]. This led us to conclude that the migration of individual invasive cells beyond the prostatic stroma has many barriers for a successful metastasis (Figure 1).

**Figure 1.**
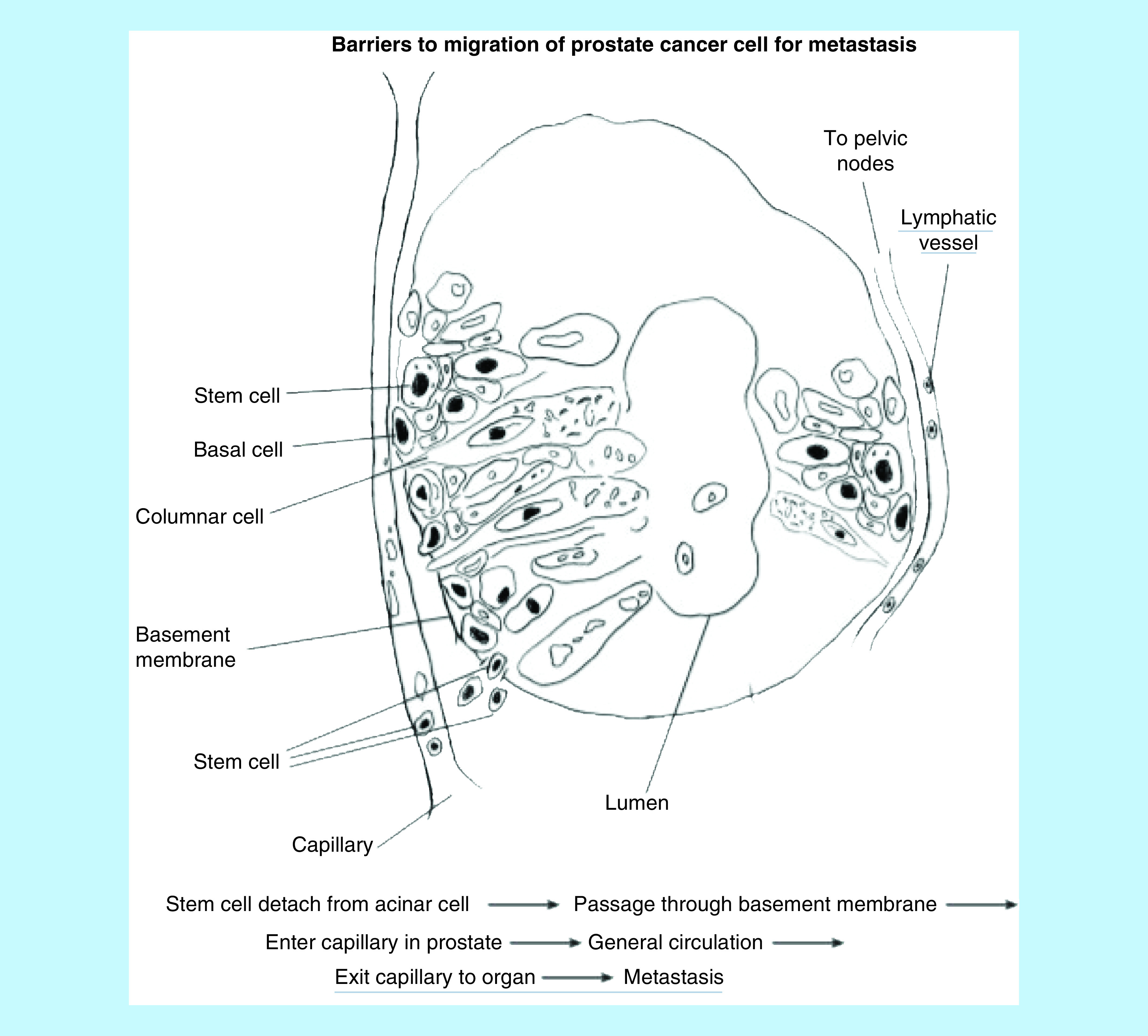
Diagrammatic figure illustrates prostatic acinus and adjacent capillary and lymphatic vessels. Diagram shows barriers to metastasis of prostate cancer cells. Each barrier requires protease(s) to lyse the membrane.

Recent studies by Wyatt *et al.* highlighted the presence of circulating DNA, which was matched with prostate biopsy studies visualized by light microscopy [21]. They suggested that DNA can be used as biomarkers [21]. Recently, Weidle *et al.* identified the functional role of metastasis-related micro-RNAs in PC [22]. This led us to hypothesize that nuclear chromatin harboring mutated DNA/genes in the nuclei of dedifferentiated columnar/cuboidal cells can readily pass through many barriers to establish metastasis in other organs. In contrast, passage of individual cancer cells beyond prostatic stroma has many barriers to reach the metastatic site(s). We have tested our hypothesis in small numbers of untreated and diethylstilbestrol (DES) treated in PC by transmission electron microscopy (TEM).

## Materials & methods

Former Veterans Affairs Medical Center (VAMC; MN, USA) urology surgeon, Dr Clyde E Blackard and his associates, selected patients for biopsy and/or radical prostatectomy. Patients were not treated with any hormone therapy or chemotherapy prior to biopsy and prostatectomy. Prostate specimens were submitted to the Pathology Service of Minneapolis VAMC and specimens not used in diagnosis were collected for research between 1972 and 1975. Tissue samples were embedded in Epon 812 and stored in our laboratory. Prostate samples were obtained following the approval of the institutional review board guidelines in place at the VA and the University of Minnesota (MN, USA). No University of Minnesota specimens were used in this study.

We received 13 untreated samples, four BPH and eight DES alone or, DES plus Provera-treated specimens. We have published dates, stages of cancer, treatments, living and death status of PC previously [14] thus, they are not repeated here. We collected prostatectomy and/or biopsy tissue specimens which were fixed for 2 h in a combination of 2% paraformaldehyde and/or 3% glutaraldehyde in a 0.1 M phosphate buffer at pH 7.3. Prostate specimens were washed in the buffer and postfixed in 1 to 2% buffered osmium-tetroxide, washed again, dehydrated in graded ethanol and embedded in Epon 812, as previously described previously [14,15,20,23,24]. Blocks were trimmed for thick and thin sections using a Reichert-Jung microtome. Thin sections (approximately 400–500 angstrom) were mounted on copper grids, stained with a combination of lead citrate and uranyl acetate, and examined with RCA EMU 3 or 4 electron microscopes, as detailed [14,15,23,24]. Reynolds has previously demonstrated that lead citrate was an electron-opaque stain [25]. Clinical details of untreated and DES-treated patients were previously published [14]. The age of untreated patients ranged from 58 to 79 years with a mean ± standard error of the mean of 70.54 ± 3.60. Range of DES-treated cases varied from 37 days to 18 years and 9 days [14]. The age of DES-treated patients ranged from 53 to 86 years, with a mean ± standard error of 69.37 ± 2.83 years. Sections were graded by Drs Donald F Gleason and Nancy A Staley, former staff pathologists at the Minneapolis VAMC. Patients had PC with pathological grades III and IV tumors, which are comparable to Gleason histological scores 6 to 10 [16,17]. Clinical stages were B, C and D [26].

## Results

**Figure 2.**
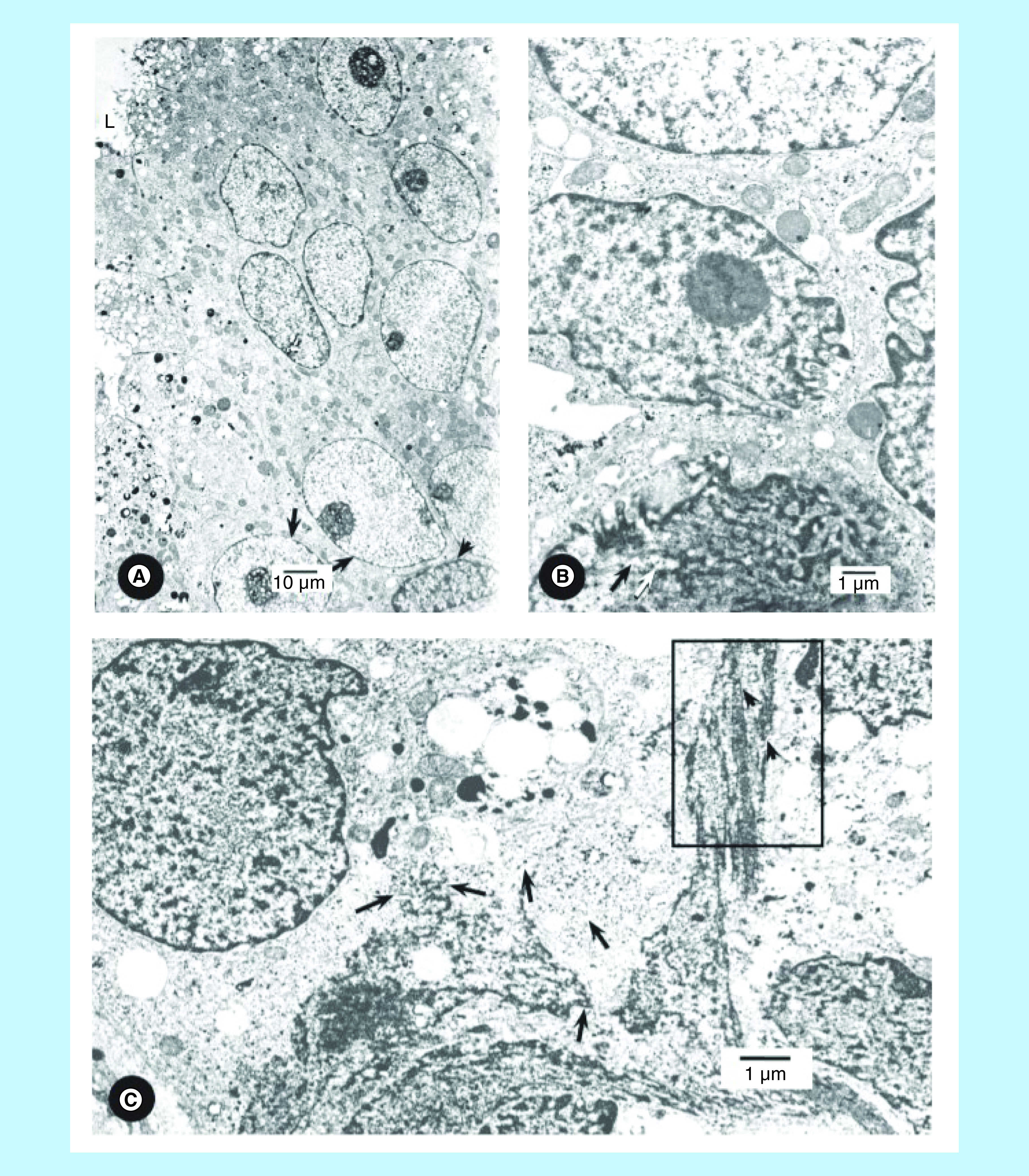
Composite figures of metastatic and non-metastatic nuclei. **(A)** Micrograph shows basal cell with a spindle-shaped nucleus (arrow head) and basally located stem cells (arrows) with intact nuclear membranes, prominent nucleoli, nuclear chromatin, mitochondria, few ribosomes indicating that stem cells are undifferentiated (poorly differentiated). Oblique sections show the basal and stem cells and some partially differentiated columnar/cuboidal cells with secretory granules and acinar lumen (L). Nuclei are oval to elongated, but not pleomorphic in acinar cells (untreated patient #110). Bar shows magnification. **(B)** Figure illustrates four nuclei of columnar/cuboidal cells. mitochondria, ribosomes and some secretory granules, all of them are usually found in dedifferentiated cells. The nucleus at the top of the micrograph shows smooth nuclear membrane associated with heterochromatin and chromatin. Another nucleus shows plasticity in the nuclear membrane as illustrated by folds whereas the other end of this nucleus is still smooth. Portion of another nucleus shows several folds. The nucleus at the bottom of the figure is completely pleomorphic. Some chromatin electron dense molecules have been released in the cytoplasm (arrow in the boxed area). Taken together, these four nuclei illustrate development of nuclear plasticity. (untreated patient #117). The bar shows magnification. **(C)** Figure illustrates a nucleus with intact nuclear membrane and condensed chromatin and heterochromatin and a set of three nuclei which have lost their shape and boundary between nuclear membranes and cytoplasm. Heterochromatin and chromatin appear as electron dense molecules (arrows). Portions of intermediate filaments are illustrated (arrow heads, area enclosed by a rectangle) as show n in Figure 3D. (untreated patient #114). The bar shows magnification.

**Figure 3.**
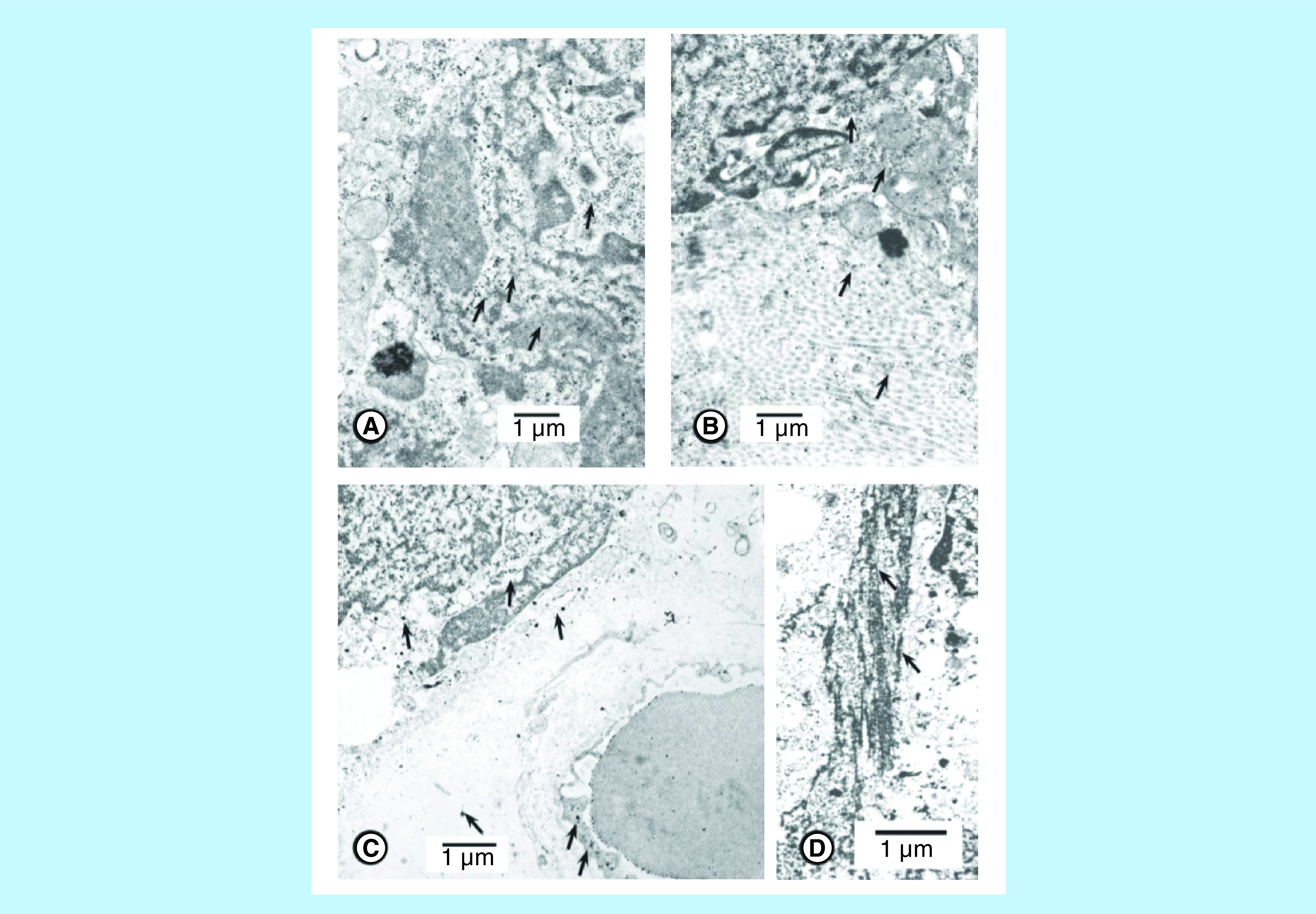
Nuclei of metastatic cells showing electron dense molecules in several compartments. **(A)** This micrograph illustrates an advanced stage of nuclear plasticity. Aggregates of nuclear chromatin/DNA appear as clusters of discrete electron dense molecules (arrows) in nucleus and cytoplasm. (untreated patient #110). The bar shows magnification. **(B)** A metastatic cell with pleomorphic nucleus in the stroma is surrounded by collagen fibers. The nucleus illustrates condensation of chromatin as electron dense molecules in cytoplasm and on collagen fibers (arrows). (untreated patient #110). The bar shows magnification. **(C)** Pleomorphic nuclei illustrates electron dense molecules. These molecules are in stroma, in capillary, capillary endothelium (arrows) and on the red cell surface. Arrows indicate that some electron dense molecules have been transported to the stroma, capillary endothelium and red cell. (untreated patient #114). The bar shows magnification. **(D)** Details of intermediate filaments shown in (Figure 2C). The figure illustrates that electron dense molecules are associated with intermediate filaments (arrows). The bar shows approximate magnification.

The prostatic stem cell has a rounded nucleus, prominent nucleolus, intact nuclear membrane, few ribosomes and small mitochondria (Figure 2A). Columnar/cuboidal cell is a lineage of stem cells and has elongated nuclei in most cancer cells and pleomorphic nuclei in some cancer cells [14,15]. The benign prostate cells do not have pleomorphic nuclei. Cuboidal/columnar cells have secretory granules, mitochondria and a portion of acinar lumen in oblique sections (Figure 2A). Secretory cells are differentiated cells whereas stem cells are poorly differentiated and have relatively few cytoplasmic organelles (Figure 2A). Inner nuclear membranes of some columnar/cuboidal cells provide a platform for anchoring intermediate filaments (Figure 2C). The inner nuclear membrane also provides areas for binding proteins for chromatin/DNA. The intermediate filaments play a role in organization of stem cell chromatin and heterochromatin and gene expression [31,32]. In contrast to the nuclei of benign prostate and BPH cells, nuclei of some cancer cells lose shape and develop plasticity (or become pleomorphic) (Figure 2B). The loss of lamins and intermediate filaments results in nuclear plasticity in some columnar/cuboidal cells (Figure 2B). The nucleus at the top of the micrograph highlights that heterochromatin is associated with the nuclear membrane and chromatin is inside the nucleus. Another nucleus shows plasticity at one end by illustrating folds in the nuclear membrane whereas the other end of this nucleus is relatively smooth. This nucleus has a prominent nucleolus. A portion of another nucleus shows folds in the nuclear membranes. The nucleus at the bottom of the micrograph is completely pleomorphic and illustrates numerous folds and condensed nuclear material (Figure 2B). Taken together, these four nuclei illustrate the development of progressive nuclear plasticity. Micrograph also illustrates a few nuclear folds, secretory granules and vacuoles and mitochondria whereas the other portion of the micrograph illustrates that nuclear membranes are totally pleomorphic and the boundary between nuclear membrane and cytoplasm is lost (Figure 2C). This releases nuclear material from the confines of the nuclear membrane to cytoplasm. A portion of a nuclear membrane with its attached intermediate filaments is demonstrated, and has also been reported by others [27,28]. The nucleus demonstrates condensed heterochromatin and chromatin. The organized structure of the nucleus is lost whereas cytoplasm still illustrates secretory granules, mitochondria and vacuoles. This brings chromatin/DNA and cytoplasm in a single compartment resulting in intermingling of nuclear contents with cytoplasmic organelles. Electron dense molecules of chromatin and/or heterochromatin are released into the cytoplasm. Intermediate filaments are still attached to the nuclear membranes (Figure 2C). Chromatin harboring DNA/genes appear as electron dense molecules. Lead citrate stains basic proteins, which bind to the DNA, producing electron dense (opaque) molecules [14,15,29]. The latter are illustrated in the nucleus and adjoining cytoplasm (Figure 3A). Another micrograph identified a part of an invasive cell nucleus with electron dense molecules, which are also distributed over collagen fibers (Figure 3B). Electron dense molecules are illustrated within and outside the nucleus (Figure 3C). Some electron dense molecules are observed in stroma between capillary and acinar cells and in capillary endothelium and on red cell surfaces (Figure 3C). Figure 3D illustrates electron dense molecules that are associated with intermediate filaments.

**Figure 4.**
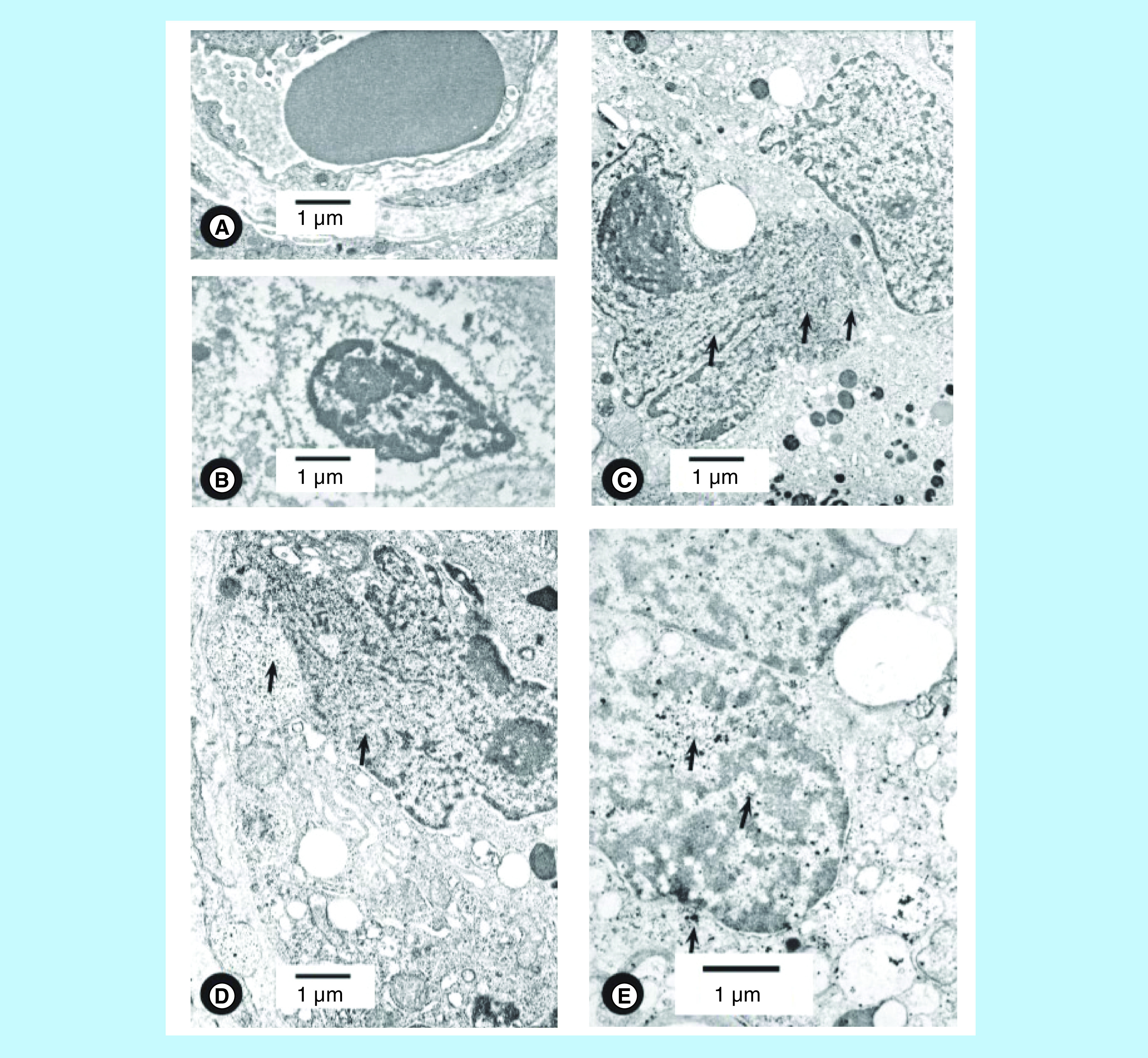
Nuclei of metastatic cells in treated cases. **(A)** A capillary with red blood cell, endothelium, stromal connective tissue and a portion of acinar cells. Capillary was not near any pleomorphic nucleus and does not show electron dense molecules in stroma and capillary endothelium. DES treated for 37 days (patient #118). The bar shows magnification. **(B)** Figure illustrates cell death with pyknotic nucleus and cytoplasm that has lost most of its organelles. Acinar lumen contained sloughed cytoplasmic portions. Pyknotic nucleus has condensed nuclear material and thickened nuclear membranes. Nucleus still contains nucleolus. Adjacent columnar/cuboidal cells did not show any signs of degeneration in nuclei and cytoplasm. This patient was treated with DES for 37 days prior to biopsy (patient #118). The bar shows magnification. **(C)** A light microscope figure of an acinus shows a migrating invasive/stem cell to stroma (arrow). Another arrow indicates acinar cells in stroma. Acinar lumen has several sloughed cells in lumen. The bar shows magnification. **(D)** In a cell, nuclear plasticity is illustrated by loss of the nuclear membranes. Some electron dense molecules (arrows) are present in the nucleus and cytoplasm. This nucleus has a large nucleolus. This patient was treated with DES for 37 days prior to biopsy (patient #118). The bar shows magnification. **(E)** Figure illustrates portions of two nuclei with condensed chromatin and heterochromatin. Electron dense molecules inside the nucleus intermingle with cytoplasmic organelles. Some of the dense molecules are present in cytoplasm (arrows) and in the nucleus area. This patient was treated with DES for 18 years and 9 days prior to biopsy (patient #104). The bar shows magnification. DES: Diethylstilbestrol.

The nuclear plasticity was also observed in DES-treated PC, but not in benign (normal) prostate and BPH [14,23]. In DES-treated cases, chromatin/DNA appeared as electron dense molecules which were released from the nucleus to cytoplasm much as in untreated cases (Figure 4D). Nucleolus was present in DES-treated cases. Nuclear membranes in adjacent acinar cells did not show plasticity (Figure 4A). Metastatic cell nucleus is distinctly different from dying cell (cell death). Cell death has condensed nuclear chromatin and heterochromatin and degenerated cytoplasmic organelles (Figure 4B). Adjacent acinar cells had not degenerated and have cytoplasmic organelles and nuclei comparable to those observed in (Figure 2A). The loss of nuclear membrane between nucleus and cytoplasm allows release of electron dense molecules from the confines of nuclear membranes into cytoplasm then in stroma and finally in nearby circulation. These molecules are carried to the capillary as highlighted by a series of micrographs (Figures 3A–C & 4C–E) and presumably to metastatic sites. Once in circulation, electron dense molecules can reach and colonize several organs (such as liver, lung, pelvic bones and/or brain). We have not studied lymphatics for the presence or absence of electron dense molecules.

## Discussion

Several studies have highlighted that DNA is shed into the bloodstream of advanced metastatic cancer and castration-resistant PC (CRPC) [21,22,30]. Circulating DNA can be used as a marker [21,22,30]. CRPC is a uniformly fatal disease [15,21]. These studies did not identify (or categorize) metastatic cells. We have demonstrated that nuclei of dedifferentiated cancerous columnar/cuboidal are involved in metastasis. Our electron microscopic analysis has shown that the metastatic cell nucleus is identified by nuclear plasticity (pleomorphic) nucleus, loss of nuclear membranes, loss of boundary between nucleus and cytoplasm and, formation of electron dense molecules of chromatin/DNA. All of these features are found only in metastatic cells and not in stromal invasive cells, the benign prostate and BPH. The presence of one or two features is inadequate to identify metastatic cells in tissue sections. This also led to further investigation of the most important features that can be utilized in diagnosis of metastatic cancer in tissue sections. The lack of boundary between nucleus and cytoplasm with the distribution of electron dense molecules are the most important features of metastatic cells. This is also supported by other studies that have highlighted that separation of nuclear and cytoplasmic compartments is critical for the functioning of cells in benign prostate and PC and other cancers [31,32]. The loss of lamins and intermediate filaments leads to nuclear plasticity of columnar/cuboidal cells [33–35]. We conclude that the lack of boundary between nucleus and cytoplasm is the single most important feature of a metastatic cell. At the present, electron microscopy is the best approach for identifying metastatic cells. Metastatic cells can be identified at light microscopy level using special stains. Our analysis of metastasis in the prostate contrasts with the numerous previous studies showing that individual cancer cells migrate to produce distant organ metastasis [1–6].

The benign prostate and PC and the benign breast and its cancer are regulated by varying amounts of steroid hormones – testosterone and estrogen – and their receptors [14,15,29,36–39]. Both of these cancers develop treatment resistance [29,36]. After studying 735 breast cancer cases Dr Stephen Paget developed his hypothesis, ‘seed and soil hypothesis’, which explained metastasis [40]. His hypothesis has endured scrutiny of over 130 years, and it is still valid in spite of the paucity of information on DNA/genes at the time. In the current study, we have identified morphological differences in invasive and metastatic cells. Metastatic cell nuclear chromatin/DNA functions as the seed and metastatic sites (such as liver, lungs, brain) function as soil. Small molecules, such as chromatin/DNA can readily pass many compartments (see result section), as can nutrients, metabolites, viruses, bacteria. Small molecules readily move in and out of cells, unlike individual cancer cells. We have, however, not shown the presence of chromatin/DNA (electron dense molecules) at metastatic sites but have provided morphological evidence that these molecules reach the capillary and red cell surface. In contrast, individual cancer cells face many barriers (Figure 1). Since our morphological study is based upon a small number of samples, it needs to be confirmed by others.

In conclusion, PC has at least two subpopulations of cells, invasive and metastatic cells. Since cells in metastasis and invasion differ, their genes ought to differ. We suggest that there are site-specific genes for metastasis in PC (e.g., liver, lungs, brain or pelvic bones) and in other solid organ cancers. Alternatively, there is a single gene or a group of related genes that are responsible for metastasis to several sites.

## Future perspective

The selection of metastatic site(s) is a random and/or semi random process. For example, PC usually metastasizes to pelvic bones, liver, lungs and brain. We postulate that the mutated PC nuclear DNA enters the nuclei of the host (e.g., liver) cells and induces them to produce PC cells. Mutated genes have a proliferative advantage whereas nonmutated genes do not. Presence of mutated prostate genes, especially in aggressive CRPC, in host cells can also induce some liver cell genes to proliferate, resulting in liver cancer. We have not shown in this study, but it would suggest the presence of metastatic PC in liver and liver cancer in liver. While metastatic PC is treated, the liver cancer remains untreated. Both types of cancers need to be treated for a successful outcome of metastatic disease. A similar scenario probably exists for metastasis in PC (e.g., lungs, pelvic bones and/or brain). A similar case can be made for breast cancer metastasis in liver, lung and for other solid organ cancers. Each cancer needs to be explored separately. Our study provides some of the reasons for the failure of treatments for metastatic PCs and other solid organ cancers. This also explains why the efforts of so many scientists and clinicians have failed to successfully treat metastatic cancers. Our idea can be readily assessed by using concurrent localization of markers for prostate and liver cancers. Our idea also needs to be explored further.

**Summary points**

- An early diagnosis of metastasis would prevent many deaths in prostate cancer and also in breast and other cancers.
- Identification of metastatic cell nucleus is not possible in the homogenized tissues.
- We have identified the metastatic cell nucleus in prostate cancer tissue sections.
- Metastatic cells are dedifferentiated columnar/cuboidal prostate cells.
- Loss of nuclear membranes between the nucleus and cytoplasm is a critical feature.
- Electron dense chromatin (DNA/genes) molecules are important features of the metastatic nucleus.
- Morphological differences in invasive and metastatic cells are probably due to their differences in genes.
